# Availability and use of telehealth services among patients with ADRD enrolled in traditional Medicare vs. Medicare advantage during the COVID-19 pandemic

**DOI:** 10.3389/fpubh.2024.1346293

**Published:** 2024-02-27

**Authors:** Nianyang Wang, Melvin Seale, Jie Chen

**Affiliations:** Department of Health Policy and Management, University of Maryland School of Public Health, College Park, MD, United States

**Keywords:** telehealth, Alzheimer’s disease and related dementias, Medicare advantage, COVID-19, racial disparities

## Abstract

**Background:**

The objective of this study was to examine differences in availability and use of telehealth services among Medicare enrollees according to Alzheimer’s disease and related dementias (ADRD) status and enrollment in Medicare Advantage (MA) versus Traditional Medicare (TM) during the period surrounding the COVID-19 pandemic.

**Methods:**

This was a retrospective cross-sectional analysis of data from community-dwelling MA and TM enrollees with and without ADRD from the Medicare Current Beneficiary Survey (MCBS) Fall 2020 and Winter 2021 COVID-19 Supplement Public Use Files. We examined self-reported availability of telehealth service before and during the COVID-19 pandemic and use of telehealth services during COVID-19. We analyzed marginal effects under multivariable logistic regression.

**Results:**

There were 13,700 beneficiaries with full-year enrollment in MA (6,046) or TM (7,724), 518 with ADRD and 13,252 without ADRD. Telehealth availability during COVID-19 was positively associated with having a higher income (2.81 pp. [percentage points]; 95% CI: 0.57, 5.06), having internet access (7.81 pp.; 95% CI: 4.96, 10.66), and owning telehealth-related technology (3.86; 95% CI: 1.36, 6.37); it was negatively associated with being of Black Non-Hispanic ethnicity (−8.51 pp.; 95% CI: −12.31, −4.71) and living in a non-metro area (−8.94 pp.; 95% CI: −13.29, −4.59). Telehealth availability before COVID-19 was positively associated with being of Black Non-Hispanic ethnicity (9.34 pp.; 95% CI: 3.74, 14.94) and with enrollment in MA (4.72 pp.; 95% CI: 1.63, 7.82); it was negatively associated having dual-eligibility (−5.59 pp.; 95% CI: −9.91, −1.26). Telehealth use was positively associated with being of Black Non-Hispanic ethnicity (6.47 pp.; 95% CI: 2.92, 10.01); it was negatively associated with falling into the age group of 75+ years (−4.98 pp.; 95% CI: −7.27, −2.69) and with being female (−4.98 pp.; 95% CI: −7.27, −2.69).

**Conclusion:**

Telehealth services were available to and used by Medicare enrollees with ADRD to a similar extent compared to their non-ADRD counterparts. Telehealth services were available to MA enrollees to a greater extent before COVID-19 but not during COVID-19, and this group did not use telehealth services more than TM enrollees during COVID-19.

## 1 Introduction

Alzheimer’s disease and related dementias (ADRD) are brain diseases that result from progressive neuron damage; they affect cognitive function, language skills, and memory, with no currently known cure (1). The number of adults with ADRD in the United States is expected to increase from 6.7 million in 2023 to 13.8 million by 2060 (1). Patients with ADRD use health services at a high rate, with an average of 6.82 to 10.18 physician office visits per year (2). When the COVID-19 pandemic arrived in the United States in 2020, patients with ADRD faced greater disruptions in their routine healthcare than their non-ADRD counterparts (3).

Telehealth became a major form of healthcare provision during the COVID-19 pandemic due to the risk of contracting COVID-19 if meeting in person, with the CDC reporting a 154% increase in telehealth use in the last week of March 2020 compared to the same week in 2019 (4). While telehealth use increased for the general population during the COVID-19 pandemic, there may be barriers to telehealth access and use among vulnerable populations, such as older adults living at home and those with ADRD (5).

Medicare is the health insurance program that is provided to adults above the age of 65 years or those with debilitating disabilities in the United States. Medicare enrollees have two options to choose from: a fee-for-service option, also known as Traditional Medicare (TM), or a managed care option known as Medicare Advantage (MA). MA may be more efficient in containing health expenditure and covering additional services such as internet access (6–9). The Creating High-Quality Results and Outcomes Necessary to Improve Chronic (CHRONIC) Care Act of 2017 provided a provision for MA plans to cover social determinants of health, such as internet costs, starting in 2020, which coincidentally aligned with the beginning of the COVID-19 pandemic in the US (10, 11).

Before the COVID-19 pandemic, TM covered telehealth only for rural areas, and patients could not use telehealth at home but rather had to go to an approved facility to receive telehealth services, while MA plans had more flexibility to offer telehealth coverage without restrictions (12). On 6 March 2020, the Centers for Medicare & Medicaid Services (CMS) released a 1,135 Waiver by authority of the Coronavirus Preparedness and Response Supplemental Appropriations Act, which expanded telehealth access for TM enrollees (13). Recent research has found that, during the COVID-19 pandemic, TM and MA enrollees had similar rates of telehealth use (44 and 45%, respectively) (12).

A previous study found no significant differences in care satisfaction or health status between TM and MA enrollees with ADRD in 2010–2016, despite MA enrollees using fewer health services (14). However, evidence comparing MA and TM enrollees with ADRD is limited, and no study has examined differences in telehealth access and use among patients with ADRD during the COVID-19 pandemic.

Guided by Penchansky and Thomas’s seminal work on the “five A’s” of access to care (availability, accessibility, accommodation, affordability, and acceptability) (15), this study examined the differences in the availability of telehealth services to Medicare enrollees before and during COVID-19 and their use of telehealth during COVID-19. We hypothesized that Medicare enrollees with ADRD would be found to use telehealth to a lesser extent than non-ADRD enrollees due to barriers to the usage of telehealth technology. We also hypothesized that MA enrollees with ADRD used telehealth services during the COVID-19 pandemic to a greater extent than TM enrollees due to the flexibility of MA plans in providing additional coverage of services such as internet access.

## 2 Methods

### 2.1 Data

This study uses the Medicare Current Beneficiary Survey (MCBS) COVID-19 Supplements for Fall 2020 and Winter 2021 (16, 17). The MCBS is an annual nationally representative survey of community-dwelling Medicare beneficiaries who are above the age of 50 years, with information on demographics, health status, and care status. The survey is conducted three times per year (in winter, covering January, February, March, and April; summer, covering May, June, July, and August; and fall, covering September, October, November, and December). The MCBS produced a series of COVID-19 Supplements providing information regarding health status and healthcare access during the COVID-19 pandemic. Data for the first COVID-19 Supplement were collected in the summer of 2020; however, we did not use this dataset, as it does not contain certain variables of interest, such as telehealth use. Informed consent was obtained at the time of the survey enrollment and was not required for this secondary data analysis.

#### 2.1.1 Sample selection

The sample for this study consisted of respondents with self-reported ADRD status, with full-year TM or MA enrollment, who knew whether their primary care provider (PCP) offered telehealth appointments. Across the combined MCBS COVID-19 Supplements for Fall 2020 and Winter 2021, data were collected from 13,770 full-year TM and MA enrollees, from a survey-weighted population of 75,141,661.

#### 2.1.2 Dependent variables

Three dependent variables were examined in this study, selected using Penchansky and Thomas’s access to care model: (15) telehealth availability before COVID-19, telehealth availability during COVID-19, and telehealth use during COVID-19. Telehealth availability before COVID-19 was defined based on whether the respondent’s PCP offered telehealth services before COVID-19. Telehealth availability during COVID-19 was defined based on whether the respondent’s PCP offered telehealth appointments at the time of the survey. Telehealth use was defined based on whether the respondent had received any telehealth visits since the previous survey wave, a period of approximately 3 months (since July 1, 2020 in the Fall 2020 survey and since November 1, 2020 in the Winter 2021 survey).

#### 2.1.3 Key independent variables

The independent variables of interest were the enrollee’s ADRD status and MA vs. TM enrollment; the interaction between ADRD status and MA enrollment was also a key effect of interest. ADRD status was defined according to whether the respondent self-reported ever having been diagnosed with ADRD. MA/TM enrollment status was determined according to whether the enrollee had either full-year MA or full-year TM enrollment; respondents with partial-year enrollment were excluded based on administrative data sources.

### 2.2 Other independent variables

Other covariates were selected based on the Andersen Behavioral Model of Health Services Use (18), their availability in the MCBS data, and recent literature on telehealth use during the COVID-19 pandemic. The Andersen model examines enrollee characteristics that are classified as relating to predisposing factors (age, sex, race and ethnicity, metro area residence, region, survey wave time period, and COVID-19 preventive behaviors), enabling resources (insurance payer, dual-eligibility status, income, speaking a language other than English at home, internet access, and telehealth-related technology access), and need characteristics (ADRD status and comorbidities).

Respondents were categorized by age as 50–64, 65–74, or 75+ years old. Sex was given as male or female. Race and ethnicity were provided under the categories White Non-Hispanic, Black Non-Hispanic, Hispanic, and Other/Unknown. Income was dichotomized as <$25,000 or ≥ $25,000 per year. Respondents’ metro residence status was defined based on the core-based statistical area (CBSA) as living in a metro or non-metro area. Region was categorized as Northeast, Midwest, South, or West. Dual-eligibility status was determined according to whether the respondent was eligible for Medicaid benefits. Speaking a language other than English at home was defined according to whether the respondent person stated that a language other than English was spoken at home. Comorbidity status are defined according to whether the respondent had ever had a heart condition, hypertension/high blood pressure, stroke, high cholesterol, cancer, osteoporosis/broken hip, emphysema/asthma/COPD (chronic obstructive pulmonary disease), diabetes/high blood sugar, depression, or a weak immune system. Survey wave was defined as Fall 2020 or Winter 2021.

We also controlled for other covariates that may predict the use of telehealth services based on Penchansky and Thomas’s model, as they related to the availability, affordability, and acceptability of digital technology. Internet access was defined according to whether the respondent had access to the internet. Having previously used video or voice calls was defined according to whether the respondent had previously used video or voice calls (for any reason) (19, 20). Telehealth-related technology access was defined according to whether the respondent owned a computer, smartphone, or tablet.

The survey also presented a set of 15 preventive behaviors and respondents were asked whether they had engaged in these behaviors due to COVID-19 (washed hands, used sanitizer, avoided touching their face, coughed/sneezed into tissue and/or sleeve, wore a facemask, cleaned common areas, avoided contact with sick people, kept 6 feet distance, avoided large groups, sheltered in place, bought extra food, bought extra cleaning supplies, bought extra medicines, consulted with their medical provider, avoided other people). The median number of preventive behaviors engaged in was 12; we created a binary index in which ≥12 was categorized as an above-median number of preventive behaviors and < 12 as a below-median number of preventive behaviors. These variables are components of accessibility and acceptability according to Penchansky and Thomas’s model; previous research has found no differences between TM and MA enrollees in the amount of preventive behavior (21).

#### 2.2.1 Statistical analysis

Logistic regression models with marginal effects were used in this study to model the outcomes while controlling for all the covariates. This was a cross-sectional analysis that used the complex survey design of the MCBS. To account for serial and inter-cluster correlation in the MCBS, balanced repeated replication (BRR) using Fay’s adjustment of 0.3 was used to estimate the variances for the standard errors. Person weights and replicate weights were pooled to account for the use of multiple waves of data. Chi-squared tests were used for categorical variables and are reported with a two-sided significance level of 0.05. All analyses were performed using Stata 15/MP and were approved by the University of Maryland Institutional Review Board.

## 3 Results

**Figure 1:**
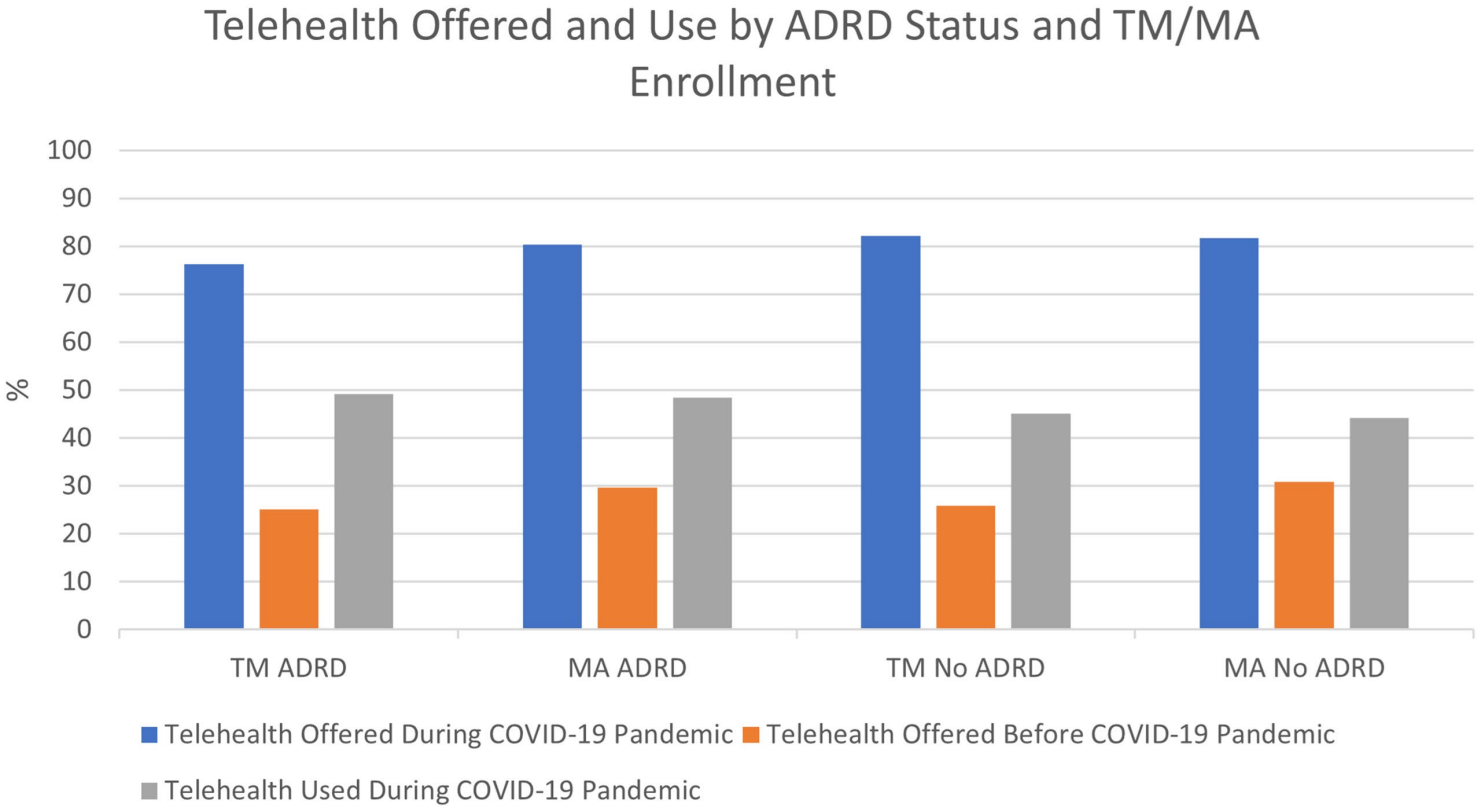
Source: 2020 Fall and 2021 Winter Medicare Current Beneficiary Survey COVID-19 Supplements. ADRD: Alzheimer’s Disease and Related Dementias, TM: Traditional Medicare, MA: Medicare Advantage.

Figure 1 shows the three outcome variables by ADRD status and TM/MA enrollment. MA enrollees were offered telehealth services at higher rates than TM enrollees before the COVID-19 pandemic (30.76% vs. 25.85%), with no significant differences between enrollees with ADRD and without ADRD. During the COVID-19 pandemic, the group with the smallest percentage of members offered telehealth services compared to all other enrollees was TM enrollees with ADRD (76.28%). A larger percentage of Medicare enrollees with ADRD used telehealth services during the COVID-19 pandemic than enrollees without ADRD (48.76% vs. 44.71%), with no significant differences between TM and MA enrollees.

Table 1 presents and compares the sample characteristics of Medicare enrollees with ADRD and those without ADRD. Enrollees with ADRD were more likely to be older (75+), to be non-White, to have an income below $25,000, to be enrolled in Medicare Advantage (46.40% vs. 39.44%), to be dual-eligible (26.30% vs. 13.84%), and to speak a language other than English at home; they were also less likely to access the internet (62.95% vs. 86.68%), to use video or voice calls (33.60% vs. 50.62%), to own telehealth-related technology (52.04% vs. 86.91%), or to engage in an above-median number of COVID-19 preventive behaviors. Enrollees with ADRD were also more likely to have ever had depression, a heart condition, hypertension, diabetes, osteoporosis/broken hip, asthma/COPD, or stroke.

**Table 1:** Sample characteristics: Medicare enrollees by Alzheimer’s disease and related dementias status.

Variable	ADRD	Non-ADRD	*p*-value
Medicare advantage	262 (46.40)	5,784 (39.44)	0.0378
Age			<0.0001
50–64 years	33 (9.66)	2,095 (13.42)	
65–74 years	75 (24.64)	5,032 (54.04)	
75+ years	410 (65.70)	6,125 (32.54)	
Female	313 (58.14)	7,209 (54.10)	0.1933
Race and ethnicity			0.0038
White non-Hispanic	341 (68.70)	9,890 (75.51)	
Black non-Hispanic	51 (10.01)	1,294 (9.77)	
Hispanic	98 (15.07)	1,375 (8.56)	
Other/unknown	28 (6.22)	693 (6.16)	
Non-metro residence	89 (14.86)	2,847 (18.25)	0.1896
Region			0.4921
Northeast	89 (14.80)	2,483 (18.56)	
Midwest	105 (20.53)	2,832 (20.60)	
South	208 (41.81)	5,047 (38.37)	
West	116 (22.86)	2,890 (22.47)	
Income ≥$25,000	264 (54.88)	8,626 (71.48)	<0.0001
Dual-eligible	163 (26.30)	2,518 (13.84)	<0.0001
Speak a language other than English at home	125 (20.39)	1,646 (11.48)	<0.0001
Winter 2021	288 (51.90)	7,224 (51.76)	0.9404
Access to internet	303 (62.95)	10,960 (86.68)	<0.0001
Use video or voice calls	170 (33.60)	6,100 (50.62)	<0.0001
Own computer/smartphone/tablet	230 (52.04)	10,953 (86.91)	<0.0001
Above-median preventive behaviors (≥12)	183 (37.57)	5,825 (44.94)	0.0034
Comorbidities			
Depression	237 (45.92)	3,523 (26.56)	<0.0001
Heart disease	229 (42.52)	4,570 (32.37)	0.0014
Cancer	116 (21.94)	2,738 (20.15)	0.4692
Hypertension	382 (71.73)	8,676 (64.21)	0.0231
Diabetes	193 (39.79)	4,372 (33.94)	0.0463
Osteoporosis/broken hip	161 (28.61)	2,834 (20.77)	0.0004
Asthma/COPD	135 (27.29)	2,628 (19.33)	0.0015
Stroke	139 (27.58)	1,242 (8.78)	<0.0001
High cholesterol	368 (72.48)	8,768 (66.26)	0.0562
Immunocompromised	94 (19.93)	2,396 (19.02)	0.7019
Total unweighted	518	13,252	13,770

Source: The Fall 2020 and Winter 2021 Medicare Current Beneficiary Survey COVID-19 Supplements.

Total weighted population: 75,141,661. ADRD, Alzheimer’s disease and related dementias; MA, Medicare advantage; COPD, chronic obstructive pulmonary disease.

Table 2 presents and compares the sample characteristics by MA vs. TM enrollment status. MA enrollees were more likely to have ADRD (3.16% vs. 2.40%), to be more than 75 years old, to be non-White, to have an income below $25,000, to live in a metro area, to be dual-eligible (18.66% vs. 11.23%), to speak a language other than English at home, and to be represented in the Winter 2021 survey; they were also less likely to access the internet (82.80% vs. 88.17%), to use video or voice calls, or to own telehealth-related technology (82.93% vs. 87.95%). MA enrollees were more likely to have hypertension, diabetes, or high cholesterol, but less likely to have a weak immune system.

**Table 2:** Sample characteristics by full-year enrollment in traditional Medicare vs. Medicare advantage.

Variable	TM	MA	*p*-value
ADRD	256 (2.40)	262 (3.16)	0.0378
Age			0.0046
50–64 years	1,286 (13.49)	842 (13.05)	
65–74 years	2,880 (54.57)	2,227 (51.23)	
75+ years	3,558 (31.94)	2,977 (35.72)	
Female	4,124 (53.08)	3,398 (55.94)	0.0585
Race and ethnicity			<0.0001
White non-Hispanic	6,139 (79.21)	4,092 (69.39)	
Black non-Hispanic	604 (7.96)	741 (12.54)	
Hispanic	554 (6.50)	919 (12.15)	
Other/unknown	427 (6.33)	294 (5.92)	
Non-metro residence	1,998 (21.32)	938 (13.34)	<0.0001
Region			0.1367
Northeast	1,503 (19.53)	1,069 (16.81)	
Midwest	1,686 (20.63)	1,251 (20.54)	
South	3,010 (39.13)	2,245 (37.46)	
West	1,525 (20.71)	1,481 (25.19)	
Income ≥$25,000	5,389 (76.50)	3,501 (62.71)	<0.0001
Dual-eligible	1,271 (11.23)	1,410 (18.66)	<0.0001
Speak a language other than English at home	728 (9.35)	1,043 (15.33)	<0.0001
Winter 2021	4,158 (50.81)	3,354 (53.21)	0.0022
Access to internet	6,516 (88.17)	4,747 (82.80)	<0.0001
Use video or voice calls	3,697 (52.63)	2,573 (46.40)	<0.0001
Own computer/smartphone/tablet	6,456 (87.95)	4,727 (82.93)	<0.0001
Above-median preventive behaviors (≥12)	3,383 (45.03)	2,625 (44.29)	0.5473
Comorbidities			
Depression	2,085 (26.59)	1,675 (27.84)	0.2359
Heart disease	2,772 (33.20)	2,027 (31.80)	0.2361
Cancer	1,637 (20.72)	1,217 (19.39)	0.1738
Hypertension	4,931 (63.02)	4,127 (66.54)	0.0089
Diabetes	2,430 (32.63)	2,135 (36.33)	0.0094
Osteoporosis/broken hip	1,678 (20.99)	1,317 (20.97)	0.9835
Asthma/COPD	1,544 (19.43)	1,219 (19.73)	0.7463
Stroke	771 (9.14)	610 (9.51)	0.6144
High cholesterol	5,012 (65.46)	4,124 (67.89)	0.0486
Immunocompromised	1,474 (20.04)	1,016 (17.52)	0.0030
Total unweighted	7,724	6,046	13,770

Source: The Fall 2020 and Winter 2021 Medicare Current Beneficiary Survey COVID-19 Supplements.

Total weighted population: 75,141,661. ADRD, Alzheimer’s disease and related dementias; TM, traditional Medicare; MA, Medicare advantage; COPD, chronic obstructive pulmonary disease.

Table 3 shows the regression for telehealth availability during and before the COVID-19 pandemic and telehealth use during the COVID-19 pandemic. The availability of telehealth services during the COVID-19 pandemic was positively associated with having an income ≥$25,000 (2.81 pp. [percentage points]; 95% CI: 0.57, 5.06), having internet access (7.81 pp.; 95% CI: 4.96, 10.66), using video or voice calls (6.28 pp.; 95% CI: 4.56, 8.00), owning a computer/smartphone/tablet (3.86 pp.; 95% CI: 1.36, 6.37), engaging in an above-median number of preventive behaviors (3.46 pp.; 95% CI: 1.77, 5.15), and being immunocompromised (2.53 pp.; 95% CI: 0.21, 4.84); it was negatively associated with being of Black Non-Hispanic ethnicity (−8.51 pp., 95% CI: −12.31, −4.71), being of “other” race and ethnicity (−5.35 pp.; 95% CI: −9.63, −1.07), living in a non-metro area (−8.94 pp.; 95% CI: −13.29, −4.59), and participating in the Winter 2021 survey (−2.77 pp.; 95% CI: −4.37, −1.16).

**Table 3:** Logistic regression: marginal effects on availability and use of telehealth services before and during COVID-19.

Variable	Telehealth availability during COVID-19			Telehealth availability before COVID-19			Telehealth use during COVID-19		
	Estimate	*p*-value	95% CI	Estimate	*p*-value	95% CI	Estimate	*p*-value	95% CI
ADRD	0.018	0.507	−0.0355, 0.0713	−0.013	0.804	−0.1147, 0.0891	−0.010	0.826	−0.1035, 0.0828
Insurance (ref = Traditional Medicare)									
Medicare advantage	0.011	0.258	−0.0084, 0.0311	0.047	0.003	0.0163, 0.0782	−0.016	0.234	−0.0412, 0.0102
ADRD × Medicare advantage	0.021	0.467	−0.0360, 0.0780	0.013	0.877	−0.1540, 0.1801	0.002	0.976	−0.1251, 0.1289
Age (ref = 50–64 years)									
65–74 years	0.033	0.028	0.0037, 0.0623	0.058	0.014	0.0120, 0.1043	−0.112	<0.001	−0.1591, −0.0640
75+ years	−0.010	0.515	−0.0420, 0.0212	0.076	0.001	0.0314, 0.1211	−0.060	0.019	−0.1104, −0.0099
Female	0.005	0.541	−0.0115, 0.0217	−0.070	<0.001	−0.0958, −0.0445	−0.050	<0.001	−0.0727, −0.0269
Race/ethnicity (ref = White NH)									
Black non-Hispanic	−0.085	<0.001	−0.1231, −0.0471	0.093	0.001	0.0374, 0.1494	0.065	<0.001	0.0292, 0.1001
Hispanic	−0.028	0.248	−0.0756, 0.0198	−0.083	0.001	−0.1339, −0.0328	0.056	0.063	−0.0030, 0.1149
Other	−0.054	0.015	−0.0963, −0.0107	0.033	0.220	−0.0202, 0.0867	0.008	0.757	−0.0436, 0.0598
Non-metro residence	−0.089	<0.001	−0.1329, −0.0459	0.000	0.999	−0.0434, 0.0433	−0.027	0.316	−0.0786, 0.0257
Region (ref = Northeast)									
Midwest	0.008	0.718	−0.0337, 0.0488	0.063	0.003	0.0212, 0.1039	−0.034	0.218	−0.0888, 0.0205
South	−0.032	0.070	−0.0659, 0.0027	0.012	0.576	−0.0298, 0.0534	0.008	0.712	−0.0354, 0.0517
West	0.034	0.155	−0.0130, 0.0809	0.141	0.001	0.0568, 0.2251	0.057	0.019	0.0096, 0.1038
Income ≥$25,000	0.028	0.015	0.0057, 0.0506	0.033	0.080	−0.0040, 0.0698	−0.015	0.371	−0.0480, 0.0181
Dual-eligible	−0.003	0.822	−0.0262, 0.0209	−0.056	0.012	−0.0991, −0.0126	0.034	0.173	−0.0152, 0.0834
Speak a language other than English at home	−0.026	0.266	−0.0708, 0.0198	0.040	0.179	−0.0189, 0.0998	−0.020	0.420	−0.0696, 0.0293
Survey wave (ref = Fall 2020)									
Winter 2021	−0.028	0.001	−0.0437, −0.0116	0.051	<0.001	0.0302, 0.0724	−0.005	0.645	−0.0262, 0.0163
Internet access	0.078	<0.001	0.0496, 0.1066	−0.038	0.172	−0.0926, 0.0168	−0.004	0.836	−0.0459, 0.0372
Use video or voice calls	0.063	<0.001	0.0456, 0.0800	−0.021	0.072	−0.0444, 0.0019	0.165	<0.001	0.1406, 0.1900
Own computer/smartphone/tablet	0.039	0.003	0.0136, 0.0637	0.011	0.640	−0.0342, 0.0554	−0.030	0.181	−0.0745, 0.0143
Above-median preventive behaviors (≥12)	0.035	<0.001	0.0177, 0.0515	0.016	0.211	−0.0090, 0.0403	0.056	<0.001	0.0351, 0.0763
Comorbidities									
Depression	0.001	0.915	−0.0184, 0.0205	−0.007	0.649	−0.0387, 0.0242	0.092	<0.001	0.0648, 0.1199
Heart disease	−0.018	0.040	−0.0349, −0.0009	−0.033	0.025	−0.0619, −0.0042	0.055	<0.001	0.0337, 0.0770
Cancer	0.010	0.337	−0.0105, 0.0303	−0.028	0.097	−0.0621, 0.0053	0.043	0.001	0.0168, 0.0685
Hypertension	−0.001	0.903	−0.0205, 0.0181	−0.020	0.094	−0.0442, 0.0035	0.033	0.018	0.0058, 0.0600
Diabetes	0.018	0.080	−0.0021, 0.0372	−0.018	0.207	−0.0454, 0.0099	0.051	<0.001	0.0262, 0.0759
Osteoporosis/broken hip	0.001	0.936	−0.0190, 0.0206	−0.012	0.469	−0.0442, 0.0205	0.044	0.001	0.0189, 0.0691
Asthma/COPD	0.010	0.330	−0.0106, 0.0313	0.008	0.624	−0.0239, 0.0397	0.041	0.008	0.0108, 0.0711
Stroke	0.008	0.530	−0.0175, 0.0338	0.041	0.061	−0.0020, 0.0835	0.015	0.477	−0.0260, 0.0551
High cholesterol	0.004	0.649	−0.0132, 0.0211	−0.019	0.196	−0.0471, 0.0098	0.011	0.331	−0.0111, 0.0327
Immunocompromised	0.025	0.033	0.0021, 0.0484	−0.005	0.742	−0.0372, 0.0266	0.102	<0.001	0.0740, 0.1306
Weighted population	75,141,661			42,441,634			60,966,722		

Source: The Fall 2020 and Winter 2021 Medicare Current Beneficiary Survey COVID-19 Supplements.

Total weighted population: 75,141,661. ADRD, Alzheimer’s disease and related dementias; TM, traditional Medicare; MA, Medicare advantage; COPD, chronic obstructive pulmonary disease.

Telehealth availability before the COVID-19 pandemic was positively associated with being of Black Non-Hispanic ethnicity (9.34 pp.; 95% CI: 3.74, 14.94), being enrolled in Medicare Advantage (4.72 pp.; 95% CI: 1.63, 7.82), falling into the age category of 65–74 years (5.82 pp.; 95% CI: 1.20, 10.43) or 75+ years (7.62 pp.; 95%CI: 3.14, 12.11), being from the Midwest (6.26 pp.; 95% CI: 2.02, 8.67) or the West (14.09 pp.; 95% CI: 5.68, 22.51), participating in the Winter 2021 survey wave (5.13 pp.; 95% CI: 3.02, 7.24); it was negatively associated with being of Hispanic ethnicity (−8.34 pp.; 95% CI: −13.39, −3.28), being female (−7.01 pp.; 95% CI: −9.58, −4.45), being dual-eligible (−5.59 pp.; 95%CI:-9.91, −1.26), and having a history of heart disease (−3.31 pp.; 95% CI:-6.19, −0.42).

Telehealth use during the COVID-19 pandemic was positively associated with being of Black Non-Hispanic ethnicity (6.47 pp.; 95% CI: 2.92, 10.01), living in the West region (5.67 pp.; 95% CI: 0.96, 10.38), using video or voice calls (16.53 pp.; 95% CI: 14.06, 19.00), engaging in an above-median number of preventive behaviors (5.57 pp.; 95% CI: 3.51, 7.63), having a history of depression (9.23 pp.; 95% CI: 6.48, 11.99), heart disease (5.53 pp.; 95% CI: 3.37, 7.70), cancer (4.26 pp.; 95% CI: 1.68, 6.85), hypertension (3.29 pp.; 95% CI: 0.58, 6.00), diabetes (5.11 pp., 95% CI: 2.62, 7.59), osteoporosis/broken hip (4.40 pp.; 95% CI: 1.89, 6.91), asthma/COPD (4.10 pp.; 95% CI: 1.08, 7.11), or being immunocompromised (10.23 pp.; 95% CI: 7.40, 13.06); it was negatively associated with falling into the age category of 65–74 years (−11.15 pp.; 95% CI: −15.91, −6.40) or 75+ years (−6.02 pp.; 95% CI: −11.04, −0.99) and with being female (−4.98 pp.; 95% CI: −7.27, −2.69).

Supplementary analysis was conducted for each of the three outcome variables while removing the use of video or voice calls and engagement in preventive behaviors as covariates; and the results of these analyses are presented in Supplementary Tables S1, S2. All variables in the model retained significance.

## 4 Discussion

This study examined the availability of telehealth services before and during the COVID-19 pandemic and telehealth use during the COVID-19 pandemic, with a focus on Medicare enrollees with respect to ADRD status and Medicare Advantage enrollment; the findings did not indicate significant differences between ADRD and non-ADRD Medicare enrollees in terms of the availability of telehealth services to these groups or their use of telehealth services. This study also showed that MA enrollees were likely to be offered telehealth services by their primary care provider before the COVID-19 pandemic, but there was no difference in availability or use of telehealth services between this group and TM enrollees during the COVID-19 pandemic.

These results suggest that policy reforms such as the CMS 1135 waivers to increase telehealth coverage for TM enrollees may have effectively addressed the TM/MA disparity in telehealth availability that was present before the COVID-19 pandemic. This notion is also supported by evidence of a sharp increase in telehealth visits among TM enrollees, from 13,000 beneficiaries receiving telehealth visits during a week before COVID-19 to 1.7 million during the last week of April 2020 (22). Another finding was that MA enrollees reported lower rates of internet access and ownership of telehealth-related technology, despite reforms such as the CHRONIC Care Act. Further analysis can examine whether reforms such as the CHRONIC Care Act are functioning to achieve their desired outcomes of improving social determinants of health for MA enrollees and whether there were challenges in implementing the CHRONIC Care Act during the COVID-19 pandemic.

While ADRD was not a significant factor in telehealth use, other comorbidities such as depression and diabetes were significantly associated with greater use of telehealth services. These comorbidities have higher prevalence in the ADRD population as compared to the non-ADRD population, which means that many patients with ADRD have co-existing conditions that need to be treated alongside ADRD to ensure good health outcomes and quality of life. In particular, depression was present in almost half of the ADRD sample and has been identified as a modifiable risk factor for ADRD (23); therefore, it is crucial to ensure that medical services such as telehealth services are available to treat both conditions. The COVID-19 pandemic may have exacerbated both ADRD and depression symptoms due to social distancing and isolation, while provision of telehealth can help improve the management of both conditions by providing virtual health services and patient monitoring when in-person services are difficult to access (24). As a result of accessing telehealth services, patients with ADRD may be less stressed about the risk of contracting COVID-19 by seeing an in-person provider and the burden of transportation for patients and their caregivers is reduced (25).

Race and ethnicity was a significant factor for all three outcomes, with Black Non-Hispanic Medicare enrollees being less likely to be offered telehealth services during the COVID-19 pandemic, more likely to be offered telehealth services before the COVID-19 pandemic, and more likely to use telehealth services during COVID-19 compared to White Non-Hispanic enrollees. These results may seem contradictory; however, they support previous research that shows that telehealth services were available to Black Non-Hispanic Medicare enrollees at higher rates than to White Non-Hispanic enrollees before COVID-19, but that during the COVID-19 pandemic, telehealth coverage subsequently increased the most for White Non-Hispanic enrollees and increased the least for Black Non-Hispanic enrollees, leading to a disparity in telehealth coverage (26). Previous research also shows that, despite telehealth services being less available to them during COVID-19, Black Medicare enrollees were more likely to use telehealth services during COVID-19 than White enrollees (12), while other research shows that Black patients were more likely to use audio-only telehealth services compared to video visits during COVID-19 (27). Further research could examine satisfaction with telehealth services and types of telehealth services used according to race and ethnicity to further examine disparities in telehealth access and quality.

Previous findings have shown that the individuals enrolled in MA are more likely to be dually enrolled in Medicaid and also more likely to be Black compared to individuals enrolled in TM (28), which we also observed in our sample. Despite differences in population characteristics, a systematic review of the literature on MA vs. TM enrollees indicated that MA enrollees had better quality of health, better health outcomes, and lower cost of care compared to TM enrollees (29).

Our study has several limitations. First, the study only included data on community-dwelling Medicare enrollees and excluded institutionalized enrollees. Previous research shows that 65% of older adults with ADRD live in the community compared to 98% of their non-ADRD counterparts (1); however, this exclusion could be regarded as a strength, as it allowed us to focus on a specific and comparable group. In addition, telehealth services were not differentiated according to whether they were provided through voice or video calls. Another limitation of this study is that we did not track switching of Medicare plans between TM and MA. Previous research as part of the 2006–2012 MCBS has shown that newly diagnosed patients with ADRD switched from TM to MA at high rates and switched away from MA plans at low rates (30), while more recent data have shown that MA enrollees with ADRD are more likely to unenroll into TM than MA enrollees without ADRD, which may indicate that MA is not meeting the medical needs of all patients with ADRD (31). This study used the MCBS COVID-19 Supplement PUFs, which were limited in the scope of available study variables compared to the MCBS Limited Data Set (LDS) (32). Further analysis of the MCBS COVID-19 Supplements linked with the MCBS LDS files could enable examination of geographic variables, such as county-level MA enrollment, as a measure of possible advantageous selection in MA (14). Finally, there is also a possibility of undercounting ADRD in survey data, as previous research has shown that improvements in the identification of neuropsychiatric disorders can be achieved by combining survey data with claims data (33).

## 5 Conclusion

Telehealth emerged as a crucial form of healthcare during the COVID-19 pandemic, but it is one that may not benefit all groups equally, potentially disadvantaging those such as patients with ADRD. This study shows that Medicare beneficiaries with ADRD did not encounter significant differences in the availability of telehealth services or make use of these services to a different extent compared to their non-ADRD counterparts. Further analysis could show changes over time in how Medicare enrollees with ADRD fared during the COVID-19 pandemic.

## Data availability statement

The original contributions presented in the study are included in the article/Supplementary material, further inquiries can be directed to the corresponding author.

## Ethics statement

Ethical approval was not required for the study involving humans in accordance with the local legislation and institutional requirements. Written informed consent to participate in this study was not required from the participants or the participants’ legal guardians/next of kin in accordance with the national legislation and the institutional requirements.

## Author contributions

NW: Conceptualization, Data curation, Formal analysis, Investigation, Methodology, Project administration, Resources, Writing – original draft, Writing – review & editing, Software, Visualization. MS: Supervision, Validation, Writing – review & editing. JC: Funding acquisition, Supervision, Validation, Writing – review & editing.
