# Supplementary material for: Availability and use of telehealth services among patients with ADRD enrolled in traditional Medicare vs. Medicare advantage during the COVID-19 pandemic
Source: Front Public Health. 2024 Feb 27;12:1346293. doi: 10.3389/fpubh.2024.1346293 (PMC10927842; doi:10.3389/fpubh.2024.1346293)
Supplement: Supplementary file 1 [file Table_1.DOCX]

| **S1**. Sensitivity Analyses of Logistic Regression Marginal Effects for Telehealth Availability Before and During COVID-19 | | | | | |  |
| --- | --- | --- | --- | --- | --- | --- |
|  | Telehealth Availability During COVID-19 | | | Telehealth Availability Before COVID-19 | | |
| Variable | Estimate | P-value | 95% CI | Estimate | P-value | 95% CI |
| ADRD | 0.01584 | 0.558 | -0.0376, 0.0692 | -0.0147 | 0.773 | -0.1156, 0.0862 |
| Insurance (ref=Traditional Medicare) | 0.00975 | 0.335 | -0.0102, 0.0297 | 0.0473 | 0.003 | 0.0165, 0.0782 |
| ADRD*Medicare Advantage | 0.02441 | 0.392 | -0.0319, 0.0808 | 0.0151 | 0.858 | -0.1525, 0.1827 |
| Age (ref=50-64 years) |  |  |  |  |  |  |
| 65-74 years | 0.03066 | 0.039 | 0.0016, 0.0598 | 0.0576 | 0.015 | 0.0116, 0.1037 |
| 75+ years | -0.01948 | 0.226 | -0.0512, 0.0122 | 0.0769 | 0.001 | 0.0320, 0.1217 |
| Female | 0.01196 | 0.160 | -0.0048, 0.0287 | -0.0694 | 0.000 | -0.0950,-0.0437 |
| Race/Ethnicity (ref=White NH) |  |  |  |  |  |  |
| Black NH | -0.07664 | 0.000 | -0.1137,-0.0395 | 0.0968 | 0.001 | 0.0405, 0.1531 |
| Hispanic | -0.02604 | 0.282 | -0.0738, 0.0218 | -0.0818 | 0.002 | -0.1330,-0.0307 |
| Other | -0.05002 | 0.020 | -0.0921,-0.0080 | 0.0350 | 0.204 | -0.0193, 0.0892 |
| Non-metro residence | -0.09627 | 0.000 | -0.1418,-0.0508 | 0.0030 | 0.892 | -0.0407, 0.0466 |
| Region (ref=Northeast) |  |  |  |  |  |  |
| Midwest | 0.00646 | 0.760 | -0.0354, 0.0483 | 0.0630 | 0.003 | 0.0213, 0.1047 |
| South | -0.03488 | 0.047 | -0.0692,-0.0005 | 0.0126 | 0.555 | -0.0296, 0.0548 |
| West | 0.03414 | 0.153 | -0.0129, 0.0812 | 0.1399 | 0.001 | 0.0553, 0.2246 |
| Income ≥$25,000 | 0.03728 | 0.002 | 0.0142, 0.0604 | 0.0293 | 0.120 | -0.0078, 0.0665 |
| Dual-eligible | -0.00204 | 0.862 | -0.0252, 0.0211 | -0.0562 | 0.012 | -0.0996,-0.0128 |
| Speak language other than English at home | -0.02444 | 0.279 | -0.0690, 0.0201 | 0.0413 | 0.173 | -0.0184, 0.1010 |
| Survey Wave (ref=Fall 2020) |  |  |  |  |  |  |
| Winter 2021 | -0.02205 | 0.007 | -0.0380,-0.0061 | 0.0508 | 0.000 | 0.0295, 0.0721 |
| Has internet access | 0.09659 | 0.000 | 0.0670, 0.1262 | -0.0430 | 0.123 | -0.0978, 0.0119 |
| Own computer/smartphone/tablet | 0.05237 | 0.000 | 0.0254, 0.0794 | 0.0070 | 0.755 | -0.0376, 0.0517 |
| Comorbidities |  |  |  |  |  |  |
| Depression | 0.00319 | 0.743 | -0.0161, 0.0225 | -0.0077 | 0.629 | -0.0391, 0.0238 |
| Heart Disease | -0.01723 | 0.050 | -0.0345, 0.0000 | -0.0330 | 0.025 | -0.0617,-0.0043 |
| Cancer | 0.00987 | 0.332 | -0.0102, 0.0300 | -0.0285 | 0.096 | -0.0622, 0.0052 |
| Hypertension | -0.00265 | 0.781 | -0.0215, 0.0162 | -0.0194 | 0.107 | -0.0430, 0.0043 |
| Diabetes | 0.01728 | 0.084 | -0.0024, 0.0370 | -0.0173 | 0.215 | -0.0447, 0.0102 |
| Osteoporosis/broke hip | 0.00129 | 0.897 | -0.0184, 0.0210 | -0.0118 | 0.476 | -0.0444, 0.0208 |
| Asthma/COPD | 0.00858 | 0.421 | -0.0125, 0.0296 | 0.0089 | 0.576 | -0.0226, 0.0404 |
| Stroke | 0.00668 | 0.606 | -0.0189, 0.0323 | 0.0412 | 0.058 | -0.0014, 0.0838 |
| High cholesterol | 0.00421 | 0.633 | -0.0132, 0.0216 | -0.0184 | 0.200 | -0.0468, 0.0099 |
| Immunocompromised | 0.03183 | 0.006 | 0.0092, 0.0544 | -0.0051 | 0.751 | -0.0366, 0.0265 |
| Weighted Population | 75,141,661 | | | 42,441,634 | | |
| Notes: Source: 2020 Fall and 2021 Winter Medicare Current Beneficiary Survey COVID-19 Supplements. | | | | |  |  |
| ADRD: Alzheimer's Disease and Related Dementias. NH: Non-Hispanic. COPD: Chronic obstructive pulmonary disease. CI: Confidence Interval. | | | | | |  |
|  |  |  |  |  |  |  |

**S2**. Sensitivity Analysis of Logistic Regression Marginal Effects for Telehealth Use During COVID-19

|  | Telehealth Use During COVID-19 | | |
| --- | --- | --- | --- |
| Variable | Estimate | P-value | 95% CI |
| ADRD | -0.0079 | 0.872 | -0.1052, 0.0893 |
| Insurance (ref=Traditional Medicare) | -0.0199 | 0.129 | -0.0457, 0.0059 |
| ADRD*Medicare Advantage | -0.0028 | 0.966 | -0.1316, 0.1260 |
| Age (ref=50-64 years) |  |  |  |
| 65-74 years | -0.1141 | 0.000 | -0.1614,-0.0669 |
| 75+ years | -0.0796 | 0.002 | -0.1291,-0.0300 |
| Female | -0.0371 | 0.002 | -0.0598,-0.0144 |
| Race/Ethnicity (ref=White NH) |  |  |  |
| Black NH | 0.0771 | 0.000 | 0.0411, 0.1131 |
| Hispanic | 0.0594 | 0.056 | -0.0017, 0.1206 |
| Other | 0.0105 | 0.700 | -0.0434, 0.0645 |
| Non-metro residence | -0.0422 | 0.116 | -0.0951, 0.0106 |
| Region (ref=Northeast) |  |  |  |
| Midwest | -0.0378 | 0.204 | -0.0965, 0.0209 |
| South | 0.0013 | 0.956 | -0.0447, 0.0473 |
| West | 0.0611 | 0.015 | 0.0119, 0.1103 |
| Income ≥$25,000 | 0.0127 | 0.437 | -0.0196, 0.0451 |
| Dual-eligible | 0.0394 | 0.120 | -0.0105, 0.0894 |
| Speak language other than English at home | -0.0199 | 0.436 | -0.0703, 0.0306 |
| Survey Wave (ref=Fall 2020) |  |  |  |
| Winter 2021 | 0.0091 | 0.391 | -0.0118, 0.0300 |
| Has internet access | 0.0365 | 0.083 | -0.0049, 0.0779 |
| Own computer/smartphone/tablet | 0.0039 | 0.862 | -0.0401, 0.0478 |
| Comorbidities |  |  |  |
| Depression | 0.0971 | 0.000 | 0.0703, 0.1239 |
| Heart Disease | 0.0581 | 0.000 | 0.0362, 0.0799 |
| Cancer | 0.0418 | 0.001 | 0.0171, 0.0665 |
| Hypertension | 0.0270 | 0.051 | -0.0001, 0.0541 |
| Diabetes | 0.0472 | 0.000 | 0.0226, 0.0718 |
| Osteoporosis/broke hip | 0.0451 | 0.001 | 0.0199, 0.0703 |
| Asthma/COPD | 0.0365 | 0.020 | 0.0060, 0.0669 |
| Stroke | 0.0112 | 0.579 | -0.0288, 0.0513 |
| High cholesterol | 0.0117 | 0.301 | -0.0106, 0.0339 |
| Immunocompromised | 0.1166 | 0.000 | 0.0881, 0.1451 |
| Weighted Population | 60,966,722 | | |

Notes: Source: 2020 Fall and 2021 Winter Medicare Current Beneficiary Survey COVID-19 Supplements.

ADRD: Alzheimer's Disease and Related Dementias. COPD: Chronic obstructive pulmonary disease.

NH: Non-Hispanic. CI: Confidence Interval.
